# Case of Bed-sore Successfully Treated by a Galvanic Current

**Published:** 1852-01

**Authors:** W. S. W. Ruschenberger

**Affiliations:** Surgeon, U. S. Navy; Philadelphia


*Case of Bed-sore successfully treated by a Galvanic Current.* By W. S. W. Ruschenberger, M. D., Surgeon, U. S. Navy.

Leopold Rogers, seaman, æt. 24, was admitted into the U. S. Naval Hospital, New York, June 3d, 1844, (then under my charge) from the U. S. ship Columbus, in which vessel he returned an invalid from the U. S. ship Congress. He suffered for a long time from syphilitic rheumatism and enlarged indurated testes. The preparations of iodine had been employed both internally and externally ; and local depletion, the deutiodide of mercury, muriate of ammonia, &c., had been resorted to without much, if any advantage. On admission, his general condition of health was feeble ; he was emaciated ; slight pressure over the epigastrium produced pain ; pulse small, and tongue coated in the centre, with clean, bright red edges. The testes were considerably enlarged, hard, tender ; and the scrotum elastic.

He suffered paroxysms of severe pain in the testes and extremities at night ; he was not able to rise from his bed. Tonics and anodynes were prescribed with a light nutritious diet, and poultices of stramonium leaves were applied to the scrotum.

On the 3d July, slight improvement of his condition was noted; paroxysms of pain less frequent, and of shorter duration ; the testes were diminished and were "less irregular" on the surface. On the 18th, a large slough had formed on the right side of the spine from pressure ; its edges were touched with nitrate of silver and a poultice applied.

23d. No improvement ; anorexia ; sloughs separating from ulcer on back.

29th. ℞. Acid. arsenios, . . . . . gr. j.
Cicutæ Ext., . . . . . gr. xxiv.
M. S. a. ft. mas. et in pil. xlviii dividend.

Cap. 1. ter die. Diet and anodynes, &c., continued.

*August 4th*, 6 o'clock P. M.—Delirious; pulse frequent and soft; twitching of muscles of neck; skin dry, but of normal temperature; frequent deglutition; "says he is quite well."

5th.—Appears to be perfectly sensible this morning. Suspend all medicine. ℞. Liq. ammoniæ acetat. ℥ss. every two hours during the day; and at bedtime, tinct. opii acetat. ʒj.

6th.—Passed a good night. The ulcer on the back is two inches in diameter; edges raised and indurated; surface cupped and sloughy, without any signs of healthy granulations.

The general condition of the patient, and his long confinement, as well as the little benefit derived from the treatment of the bed-sore which had become a source of great irritation and annoyance, led to an unfavorable prognosis. But little amelioration could be anticipated, unless the ulcer on the back could be healed.

In the autumn of 1842, I had resorted to Mr. Mansford's galvanic plates, in the treatment of a case of epilepsy, and found difficulty in preventing the surface under the silver plate from healing. I discovered, however, that by alternating the zinc and silver plates, that a discharge was kept up from both blistered surfaces, and that either would heal under the continuous application of the silver plate, in the course of a few days, while ulceration extended, seemingly, in a corresponding manner, under the zinc.

This circumstance suggested a trial of Mr. Mansford's plates in the treatment of the bed-sore in the case of Rogers. As he suffered very much from pain and swelling about the right malleolus externus, a blister two inches square was applied over it at night. On the morning of the 7th of August, the cuticle was removed, and Mr. Mansford's plates applied, the silver plate being placed over the ulcer on the back and the zinc over the ankle: the plates were about two inches in diameter; slips of buckskin were substituted for muscle, which is directed by Mr. Mansford. A full description of the apparatus and its mode of application, may be found in Professor Dunglison's very valuable work, entitled "New Remedies" under the head of "Galvanismus."

*August 11th.*—Ulcer is assuming a decidedly better aspect; granulations shooting up healthily; edges less elevated and not so thick; discharge laudable. Continue plates, changing the dressing daily. Suspend liq. ammon. acetat.; resume pills of cicuta and arsenic; anodyne at night.

15th.—The surface beneath the zinc plate has become ulcerated; apply a blister an inch square over opposite malleolus. P. M. Transfer zinc plate to newly blistered surface.

21st.—The ulceration beneath the zinc plate so much extended that the apparatus is discontinued. Simple dressings to ankles; ceratum zinc. carb. to back.

26th.—Paroxysm of severe pain in ankles last night; relieved by anodynes.

28th.—Ulcers on back healing rapidly; those over the ankles nearly well.

A tonic treatment, with nourishing diet, and subsequently syrup of sarsaparilla with iodide of iron, were continued until January 16th, 1845; when the patient was discharged from the hospital, at his own request, almost entirely restored to health.

The application of this galvanic pair was continued during fourteen days, when the ulcer on the back had improved so much, that it could be treated, in my opinion, by the usual dressings. Had it been desirable to continue the apparatus, it would have been necessary only to remove the cuticle from other points for the accommodation of the zinc plate.

The above statement is made from notes of the case made by Passed Assistant Surgeon, Jas. B. Gould, U. S. Navy.

The July number of the "Medical Examiner" for 1851, (p. 481) contains a notice of a case of "Indolent Ulcer, speedily cured by the Electric Moxa," prescribed by Mr. Bransby B. Cooper. A perusal of this notice, and of Professor May's description of a case of amputation at the hip joint, (published in "The American Journal of the Medical Sciences" for October, 1851, p. 218) in which the patient suffered from "sloughing over the sacrum, induced by pressure," reminded me of my satisfactory experiment in the case of Rogers; the above cases, in my estimation, added to its value as a single medical fact, which is perhaps worthy of record.

My friend, the late Dr. Washington of New York, at my susgestion treated a similar case with the same success, in the winter of 1845.

*Philadelphia, December*, 1851.

